# Real Time Precise Relative Positioning with Moving Multiple Reference Receivers

**DOI:** 10.3390/s18072109

**Published:** 2018-06-30

**Authors:** Hui Li, Shuang Gao, Liang Li, Chun Jia, Lin Zhao

**Affiliations:** College of Automation, Harbin Engineering University, Harbin 150001, China; lihuiheu@hotmail.com (H.L.); gaoshuang@hrbeu.edu.cn (S.G.); jiachun@hrbeu.edu.cn (C.J.); zhaolin@hrbeu.edu.cn (L.Z.)

**Keywords:** satellite positioning, moving multiple reference receivers, fused measurement, integer ambiguity resolution

## Abstract

The stationary reference receiver with precisely known coordinates is difficult to establish in some special real-time relative positioning applications. To improve the relative position estimation accuracy and the reliability simultaneously for the RTK without a precisely known reference receiver, multiple receivers mounted on a moving platform are used as the base station. A code and phase measurement fusion model is proposed to reduce the communication burden and generate measurements at any virtual position where it is inconvenient to install the GPS receiver. To keep the integer property of the ambiguity of fused phase measurements, the RTK method with the moving reference receivers is proposed by implementing the integer ambiguity transformation and error absorption strategy based on the known geometry of multiple receivers. Static and kinematic experiments were carried out to evaluate the performance of the proposed relative positioning method. When compared with the single-receiver solution, static results have shown that the proposed method can improve position accuracy by 15.9% and 15.7% for the horizontal and the vertical component, respectively. The kinematic results have shown that the proposed method can achieve position accuracy enhancement by 26.9% for the vertical component.

## 1. Introduction

Real Time Kinematic (RTK) has been proven to be an efficient and reliable technology for high-precision positioning over the past decades [1]. By resolving integer ambiguity, centimeter-level position estimation accuracy can be achieved in both real-time and post-processing modes [2,3]. Since the RTK outputs the relative position between the base station and the rover, the accurate absolute position of the rover needs to use the coordinates of a stationary reference station. Many applications require the relative position, such as the positioning of cranes on ships, aerial docking of spacecraft, formation flying, relative positioning of vehicles, fleet management, and deformation monitoring of large moving vehicles [4,5]. In such applications, it is difficult to establish a stationary reference station with accurately known coordinates. The moving reference receiver cannot give its precise position as a priori, and furthermore, as it is in motion, the measurements can be easily corrupted by, for example, multipaths [6,7]. The traditional RTK technique cannot be applied directly to moving reference receiver without precise position [4,8].

The research on moving reference receivers was started in the 1990s [9]. Binning studied the single difference positioning method for satellite to satellite navigation using simulated data [10]. Kawano used dynamic to dynamic technology based on pseudo difference for on-orbit satellite docking [11]. The idea of using multiple receivers to improve position estimation accuracy or to determine attitude was explored by several research groups [8,12,13,14,15]. Since carrier phase integer ambiguity resolution plays an important role in the precise positioning and attitude determination, there are many documented studies focusing on ambiguity resolution in the ambiguity domain [16,17,18,19]. These studies incorporate known baseline constraints in the ambiguity search algorithms to get efficient integer search strategies. Another class of studies mainly enhances the performance of float solutions of ambiguities in the positioning domain. Since multiple receivers mounted on the platform in an array of known geometry increases redundancy [20], these studies use multiple receiver configurations to strengthen the observational model [12,21,22,23]. In these studies, multiple receivers are utilized without considering the communication burden, and the baseline constraints are used to strengthen the position estimation model to improve the relative positioning performance using a stationary base station. Since multiple receiver configuration improves positioning performance efficiently, it can be also used in the base station. For fixed base stations, the technique using multiple reference receivers is known as the Network RTK (NRTK). In the NRTK technique, multiple reference receivers are stationary with precisely known coordinates, and the inter-station baseline distances in the current NRTK implementations are typically restricted to around 50 km or less [24,25]. Therefore, the high-precision network-derived atmospheric corrections in both spatial and temporal domain can be computed to correct the rover measurement error when ambiguity between reference stations are fixed [26]. However, these technologies cannot be applied in the RTK with multiple moving reference receivers. Most of the applications mentioned above are practically implemented in air or sea. They encounter three problems. First, land-based GPS stationary stations cannot be used directly, because the users are far away from land. Second, the reference receivers used are usually in motion. Third, the accurately absolute coordinates of reference receivers are difficult to determine. Therefore, modifications are required for moving RTK with multiple moving reference receivers. Luo proposed the ‘Multi-Kin’ method to process GPS observations from multiple (more than three) moving platforms simultaneously, making full use of constraints through the multiplicity of platforms to improve the ambiguity resolution [5]. The ‘Multi-Kin’ method is only suitable for applications that have multiple moving platforms, each of which is equipped with one receiver. Schrader used multiple GPS receivers and supporting electronic components to improve the accuracy of GPS data [27]. Trinklein employed truncated mean and moving average filters of the latitudes and longitudes to post process multiple moving inexpensive GPS receiver data, and yielded approximately one meter accuracy for the relative position vector [14].

In some critical real-time systems, both measurements and positioning performance can be deteriorated severely under challenging conditions, such as electromagnetic interference. In this case, multiple moving reference receiver configuration as the base station is needed to improve the situation. As we use all the measurements from multiple reference receivers, the communication burden between the reference receivers and the user has to be considered. To improve relative positioning performance without affecting the real time performance of positioning, a real time code and phase measurement fusion model is proposed. The model makes full use of known antenna geometry, and similar ionospheric and tropospheric delays to fuse the original measurements from different reference receivers on a moving platform. Moreover, to avoid destroying the integer property of ambiguity of fused phase measurements, both the integer ambiguity transformation and error absorption are applied. Errors that may be introduced in the process of generating fused measurements are evaluated. The static experiment and kinematic experiment were formed to assess the performance of proposed methodologies.

The contribution is organized as follows. In Section 2, the details of the model are outlined, including the original un-differenced phase and code observation models, measurements fusion model, integer ambiguity re-parameterization, and stochastic error model. In Section 3, experiments and analyses of the methodology are given. In Section 4, the conclusion remarks are presented.

## 2. Methodology

### 2.1. Functional Model

Considering the satellite and receiver clock biases and various propagation delays, the original un-differenced phase, and code observation models on frequency *j* can be expressed as [28,29,30]:

(1) $$P_{r,j}^{}=R_{r}^{}+\mu_{j}I_{r}^{}+T_{r}^{}+dt_{r,j}-dt_{,j}^{}+\varepsilon_{P_{r,j}^{}},$$

(2) $$\lambda_{j}\phi_{r,j}^{}=R_{r}^{}-\mu_{j}I_{r}^{}+T_{r}^{}+\lambda_{j}N_{r,j}^{}+\delta t_{r,j}-\delta t_{,j}^{}+\varepsilon_{\phi_{r,j}^{}},$$

where the subscripts *r* and *j* denotes the receiver and frequency *f_j_*, which are used to emphasize the receiver-specific and frequency-specific term, respectively. ***P****_r_*_,*j*_ = [*P_r_*_,*j*_^1^, …, *P_r_*_,*j*_*^s^*]^T^ and ***φ****_r_*_,*j*_ = [*ϕ_r_*_,*j*_^1^, …, *ϕ_r_*_,*j*_*^s^*]^T^ are the un-differenced code measurement in meters and phase measurement in unit of cycle, and the superscripts *s* denote the satellite. ***R****_r_* = [*R_r_*^1^, …, *R_r_^s^*]^T^ is geometric distance; ***I****_r_* = [*I_r_*^1^, …, *I_r_^s^*]^T^ is the ionosphere delay on frequency *f*_1_ with *u_j_* *= f*_1_^2^*/f_j_*^2^; ***T****_r_* = [*T_r_*^1^, …, *T_r_^s^*]^T^ is tropospheric delay; *dt_r_*_,*j*_ and ***dt***_,*j*_ = [*dt*_,_^1^, …, *dt*_,_*^s^*]^T^ are the receiver and satellite clock offset for code respectively, which includes clock error and frequency-dependent code biases; *δt_r_*_,*j*_ and ***δt***_,*j*_ = [*δt*_,_^1^, …, *δt*_,_*^s^*]^T^ are the receiver and satellite clock offset for phase respectively, which includes clock error and frequency-dependent phase biases; *λ_j_* is the phase wavelength and ***N****_r_*_,*j*_ = [*N_r_*_,*j*_^1^, …, *N_r_*_,*j*_*^s^*]^T^ is un-differenced integer ambiguity; *ε_Pr_*_,*j*_ is the receiver code noise in meters, including multipath error in codes; *ε_ϕr_*_,*j*_ is the receiver phase noise in meters, including phase multipath errors.

### 2.2. Measurement Fusion Model

**Figure 1:**
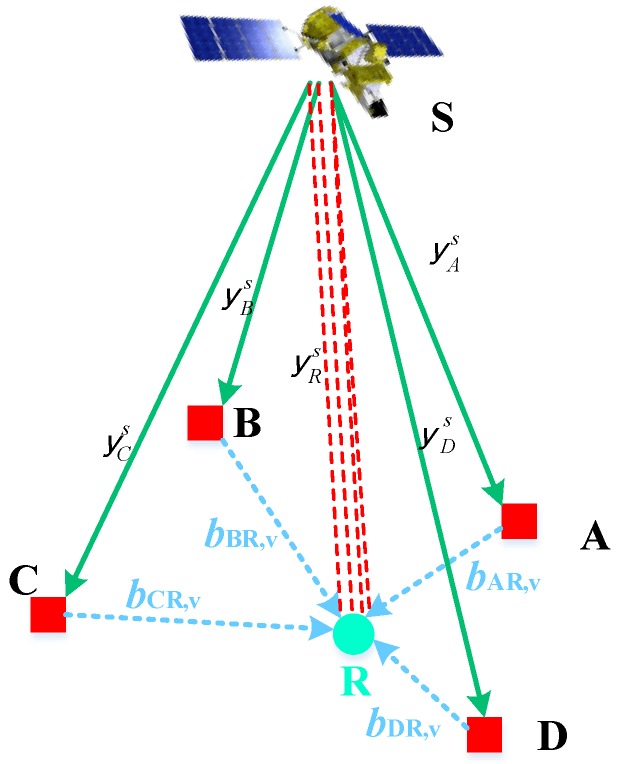
Multiple reference receiver configuration architecture.

The measurement fusion model can combine the code and phase measurements from different reference receivers to generate fused measurements at any virtual locations within the network of reference receivers. Multiple reference receivers can be deployed in different locations of the common moving platform, where there are minimal electromagnetic interferences and multipath effects. Constructing fused measurements in real time requires an accurate knowledge of the baseline lengths between the reference receivers and the position of the reference point. Figure 1 schematically depicts the measurements transferred from multiple receivers, taking four receivers as an example. We choose the point R as the location of a virtual receiver and transfer the code and phase measurements from the reference receivers to this reference point. Point A, B, C, D are reference receivers, and ***y****_r_* = [***P****_r_*, ***φ****_r_*]^T^ denotes the code and phase measurements from the reference receivers.

To generate fused measurements at the virtual point R, we need to convert the measurements from different receivers, as shown by the red dotted lines in Figure 1. As various reference receivers are installed on the same moving platform, the distances between each receiver and the point R are relatively short. Therefore, their satellite line-of-sight vectors are assumed to be parallel with each other. The projection of the baseline vector on the satellite observation vector is equal to the change of geometric distance between satellite and receiver. Taking advantage of the close distance between reference receivers, we can assume that the atmospheric delays of the satellite signals at all receivers (A, B, C, and D) are similar. Therefore, the main difference of measurement between the reference point R and each receiver is the difference of their geometric distance between satellite and receiver caused by the baseline vector. For receiver *r*, the transferred measurements at the point R can be given as:

(3) $$y_{R,r}^{}=y_{r}^{}+\Delta R_{R,r}+\varepsilon_{y_{R,r}^{}},$$

(4) $$\Delta R_{R,r}=e_{r,e}b_{Rr,e},$$

where ***y****_R_*_,*r*_ = [***P****_R_*_,*r*_, *λ****ϕ****_R_*_,*r*_]^T^, ***P****_R_*_,*r*_ = [*P_R_*_,*r*,1_, …, *P_R_*_,*r*,*n*_]^T^ and ***ϕ****_R_*_,*r*_ = [*ϕ_R_*_,*r*,1_, …, *ϕ_R_*_,*r*,*n*_]^T^ are the transferred measurements from receiver *r*. ***λ*** = [*λ*_1_, …, *λ_n_*]^T^ is the phase wavelength of different frequency. ***ε****_PR_*_,*r*_ and ***ε****_yR_*_,*r*_ = [***ε****_PR_*_,*r*_, ***ε****_ϕR_*_,*r*_]^T^ is the transferred measurements noise in meters. Δ***R****_R_*_,*r*_ is the changed geometric distance between the reference point R and receiver *r*. ***e****_r_*_,*e*_ is the unit observation vectors of receiver *r*; the subscripts *e* denote the WGS-84 ECEF coordinate frame; ***b****_Rr_*_,*e*_ is baseline between the receiver *r* and the point *R*.

Equations (1)–(4) can be used to generate the transferred measurements from receiver *r*. The fused code and phase measurements on point R can be expressed as:

(5) $$y_{R}=\sum_{r=1}^{m}\alpha_{r}y_{R,r},$$

where ***y****_R_* = [***P****_R_*, ***ϕ****_R_*]^T^, *α_r_* is weighting coefficients, and *m* denotes the number of the reference receivers.

### 2.3. Integer Ambiguity Re-Parameterization

For the code measurement, Equation (5) provides the final fused code measurements of point R. However, for the fused phase measurement, the introduction of weighting coefficients *α_r_* will destroy the ambiguity’s integer property. From Equations (2), (3) and (5) the final fused phase measurements of satellite *s* at the point R can be generated as:

(6) $$\lambda_{j}\phi_{R,j}^{s}=\rho_{R,j}^{s}+\lambda_{j}N_{R,j}^{s}+\sum_{r=1}^{m}\alpha_{r}\delta t_{r,j}-\delta t_{,j}^{s}+\varepsilon_{\phi_{R,j}},$$

(7) $$N_{R,j}^{s}=\sum_{r=1}^{m}\alpha_{r}N_{r,j}^{s},$$

(8) $$\rho_{R,j}^{s}=\sum_{r=1}^{m}\alpha_{r}\left(R_{r}^{s}+\Delta R_{R,r}^{s}-\mu_{j}I_{r}^{s}+T_{r}^{s}\right),$$

(9) $$\varepsilon_{\phi_{R,j}}=\sqrt{\sum_{r=1}^{m}{\left(\alpha_{r}\varepsilon_{\phi_{r,j}}\right)}^{2}}.$$

In Equation (6), $N_{R,j}^{s}$ is not an integer. Therefore, we need to re-parameterize the combined integer ambiguity. The multiple reference receiver configuration makes it possible to form ambiguity transfer. In this approach, the ambiguities between moving reference receivers are used. A main reference receiver needs to be chosen from all the reference receivers. Without the accurately known coordinates of the reference receivers, we use the baseline length constraints to strengthen the double-differenced (DD) phase and code measurement equations. The DD integer ambiguity among the reference receivers can be obtained reliably. Using the DD integer ambiguity, $N_{R,j}^{s}$ in Equation (6) can be expressed as:

(10) $$N_{R,j}^{s}=\sum_{r=1}^{m}\alpha_{r}N_{1r,j}^{1s}+N_{1,j}^{s}+\sum_{r=1}^{m}\alpha_{r}(N_{r,j}^{1}-N_{1,j}^{1})$$

where the subscript 1 denotes the main reference receiver and the superscripts 1 denotes the main reference satellite. The term $\alpha_{r}(N_{r,j}^{1}-N_{1,j}^{1})$ is only affected by the receiver for a fixed frequency. It has the same properties as the receiver clock offset, therefore it can be absorbed by the receiver clock offset as:

(11) $$\delta t_{R,j}=\sum_{r=1}^{m}\alpha_{r}\delta t_{r,j}+\lambda_{j}\sum_{r=1}^{m}\alpha_{r}(N_{r,j}^{1}-N_{1,j}^{1})$$

The DD integer ambiguity between reference receivers which is $N_{1r,j}^{1s}$ in Equation (10) can be calculated based on the known body-frame geometry of multiple reference receivers [22]. Therefore, the final fused phase measurements to point R can be given as:

(12) $$\lambda_{j}{\tilde{\phi }}_{R,j}^{s}=\rho_{R,j}^{s}+\lambda_{j}N_{1,j}^{s}+\delta t_{R,j}-\delta t_{,j}^{s}+\varepsilon_{\phi_{R,j}},$$

where $N_{1r,j}^{1s}$ represents the ambiguity in the fused phase measurements. It can be found that the combined integer ambiguity is not only immune to the coefficient *α_r_* as 1/*m*, but also preserves the integer property of the integer ambiguity.

### 2.4. Stochastic Error Model

Since the stochastic model plays an important role in resolving the integer ambiguity [31,32], we need to characterize the stochastic error model of the fused measurements. Based on Equations (1) and (2), we define the observation vector from each receiver ***y****_r_* = [*P_r_*_,1_, …, *P_r_*_,*n*_, *ϕ_r_*_,1_, …, *ϕ_r_*_,*n*_]^T^, where the subscripts *n* represents the number of frequency. *Q_r_*, the stochastic model of *y_r_*, can be specified by:

(13) $$Q_{r}=Q_{m}⊗Q_{w},$$

where ***Q****_m_* = blkdiag(*Q_p_*, *Q_ϕ_*) stands for the precision of phase and code measurement. ***Q****_p_* = diag(*σ_p_*_1_^2^, …, *σ_pn_*^2^) and ***Q****_ϕ_* = diag(*σ_ϕ_*_1_^2^, …, *σ_ϕn_*^2^), where *σ_p_*_*j*_^2^ and *σ_ϕ_*_*j*_^2^ are the variance of the un-differenced phase and code measurements. It is assumed that the precision of phase and code measurements are unique for both frequencies. ***Q****_w_* is the weight matrix of the un-differenced measurements. During the construction of the fused GNSS measurements, accurate knowledge of the baseline length relative to WGS-84 ECEF coordinate frame between each receiver and the virtual point R is required. If the baseline length in ECEF frame is unknown, it needs to be calculated by baseline length in the body frame and attitude in the navigation frame. Therefore, along with the GPS measurement errors of each receiver, the baseline length errors between each receiver and point R are of great importance in the generation of fused measurements. The baseline lengths in the body coordinate frame can be determined from a platform survey or other methods which are transferred into the WGS-84 ECEF coordinate frame with the knowledge of the attitude and position of the platform. Therefore, the calculation of baseline length in WGS-84 ECEF coordinate frame is coupled with uncertainties due to baseline length errors in body frame and attitude errors of the platform. An expression for baseline length ${\hat{b}}_{Rr,e}$ can be simply written as

(14) $${\hat{b}}_{Rr,e}=b_{Rr,e}+\delta b_{Rr,e},$$

in which ${\hat{b}}_{Rr,e}$ is the estimated value of baseline length used in Equation (4), which is corrupted by estimation error *δb_Rr_*_,*e*_. With the baseline length in body coordinate frame, ${\hat{b}}_{Rr,e}$ can be obtained as

(15) $${\hat{b}}_{Rr,e}={\hat{T}}_{n}^{e}{\hat{T}}_{b}^{n}{\hat{b}}_{Rr,b},$$

where the subscripts *n* and *b* denotes the navigation coordinate frame and the body coordinate frame respectively. The superscript and subscripts *e* denote the WGS-84 ECEF coordinate frame. ${\hat{T}}_{n}^{e}$ is the transformation matrix from navigation frame to WGS-84 ECEF frame and ${\hat{T}}_{b}^{n}$ is the transformation matrix from body frame to navigation frame. The hat notation indicates that the terms have nominal errors.

Due to the platform attitude errors, ${\hat{T}}_{b}^{n}$ can be expressed as

(16) $${\hat{T}}_{b}^{n}=T_{b}^{n}+CT_{b}^{n},$$

where ***C*** is the skew symmetric matrix caused by attitude errors and determined by the rotation vector of attitude errors in the navigation frame. Compared with attitude errors of the platform, the errors of the transformation matrix caused by platform position estimation error are negligible, i.e., ${\hat{T}}_{n}^{e}=T_{n}^{e}$.

Therefore, the baseline vector errors *δb_Rr_*_,*e*_ can be expressed as follows:

(17) $$\delta b_{Rr,e}=T_{n}^{e}T_{b}^{n}\delta b_{Rr,b}+T_{n}^{e}CT_{b}^{n}b_{Rr,b}+T_{n}^{e}CT_{b}^{n}\delta b_{Rr,b},$$

in which *δb_Rr_*_,*e*_ contains three parts. The first part *T_n_^e^T_b_^n^δb_Rr_*_,*b*_ in the right hand represents baseline length survey errors, which are mainly affected by the survey methods. The second part *T_n_^e^CT_b_^n^b_Rr_*_,*b*_ is determined by the attitude errors of the platform The third part couples the survey errors with attitude errors. In this paper, we assume that the attitude and baseline length survey errors are small enough, so that the third term can be neglected.

The covariance for baseline vector errors *δb_Rr_*_,*e*_ can be equated as

(18) $$Q_{b}=E(\delta b_{Rr,e}\delta b_{Rr,e}^{T})=RD(\delta b_{Rr,b})R^{T}+MD(\delta e)M^{T},$$

(19) $$M=T_{n}^{e}\left[\begin{array}{ccc} 0 & b_{Rr,n}(3) & -b_{Rr,n}(2)\\ -b_{Rr,n}(3) & 0 & b_{Rr,n}(1)\\ b_{Rr,n}(2) & -b_{Rr,n}(1) & 0 \end{array}\right],$$

(20) $$b_{Rr,n}=T_{b}^{n}b_{Rr,b},$$

where *E* and *D* are the expectation and covariance operators, respectively, and $R=T_{n}^{e}T_{b}^{n}$. The first term in the right hand is caused by baseline length errors, while the second term is caused by attitude errors. Equation (18) can be used to assess the stochastic model of the baseline errors. A characterization of baseline errors will benefit ambiguity resolution using the fused measurements. From Equations (14)–(20), it is clear that the term *δb_Rr_*_,*e*_ is caused by baseline errors in body frame and the rotation vector of attitude errors in the navigation frame. Therefore, the transferred measurement ***y****_R_*_,*r*_ in Equation (3) includes the translating error caused by *δb_Rr_*_,*e*_ in translating process. The weighting coefficients *α_r_* in Equation (5) is set to 1/*m*, which means the fused measurements are generated by calculating the mean of the transferred measurements. It is noted that when making an average operation, errors and variances are pulled down at the same time. Therefore, the measurement fusion model is helpful in diminishing the influence of the individual measurement error.

## 3. Experiment and Analysis

We have verified the proposed method by conducting the static and kinematic experiments. We extensively compared the ambiguity resolution and the positioning accuracy performance of the single receiver-antenna (SA) solution and multiple moving receiver-antenna (MA) solution. The SA solution means using a single antenna and receiver as the base station. Both experiments use dual-frequency code and phase GPS measurements. The standard deviation of un-differenced code and phase measurement is 30 cm and 3 mm respectively, by keeping measurement type weighting. It has been widely proven that the LAMBDA algorithm has the best success rate among the different integer aperture estimation methods [33]. We therefore used the LAMBDA for ambiguity resolution. Since the proposed method can mitigate the effect of measurement noise from the combination of multiple reference receivers, an optimistic threshold is preferable to prove the edge of our proposed method. The threshold for ratio test is set up to 1/2 [34,35]. The ambiguity success rates are computed by comparing the single-epoch estimated ambiguities to the reference ambiguities obtained from the whole span of data. To evaluate position estimation accuracy, the horizontal and vertical position error (HPE/VPE) are:

(21) $$HPE=\sqrt{\Delta x_{E}^{2}+\Delta x_{N}^{2}},$$

(22) $$VPE=\left|\Delta x_{U}^{}\right|,$$

where Δ*x_E_*, Δ*x_N_*, Δ*x_U_* is the relative position estimation error of east, north and up component respectively. To quantify relative position estimation precision, assuming Rayleigh and Gaussian distribution of horizontal and vertical error, Equations (23) and (24) are used to compute the 95% horizontal and vertical error bounds, where operator std() denotes standard deviation operator [36].

(23) $$\sigma_{H,95\%}=2\times \sqrt{std{\left(\Delta x_{E}^{}\right)}^{2}+std{\left(\Delta x_{N}^{}\right)}^{2}}$$

(24) $$\sigma_{V,95\%}=1.96\times std(\Delta x_{U})$$

### 3.1. Static Experiment

**Figure 2:**
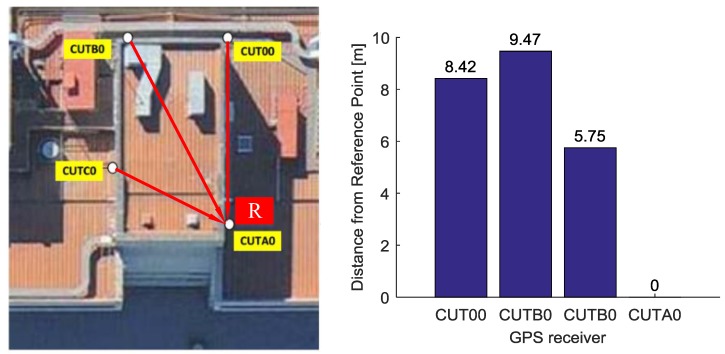
Distribution of multiple receivers. The (**Left**) panel is the location of receivers. The (**Right**) panel is the baseline between each receiver and point R.

In the experiment, the performance of real-time relative positioning using MA solution are compared with commonly used SA solutions. The data from Curtin GNSS Research Centre was used as multiple reference receiver data in the static experiment. There are four stations marked CUTB0, CUTC0, CUT00, CUTA0 on the roof of the building of Curtin University in Perth, Australia, whose locations are precisely known. Each station is equipped with a TRM59800.00-SCIS antenna (Trimble Inc., Sunnyvale, CA, USA) which is connected with TRIMBLE NETR9 receiver (Trimble Inc., Sunnyvale, CA, USA). We assumed that entire roof would act as a base station; the receivers of CUTB0, CUTC0, CUT00 are used as reference receivers. We selected the location of station CUTA0 as the virtual point. Given the coordinates of each antenna in ECEF frame, we calculated the baseline length between the multiple reference receiver antennas and the virtual point. The distribution of CUTB0, CUTC0, CUT00, CUTA0 and the calculated baseline lengths are shown in Figure 2. The details of the data used including baseline length between each reference receiver and the virtual point, the time of data collection, and the sampling rate are given in Table 1.

**Figure 3:**
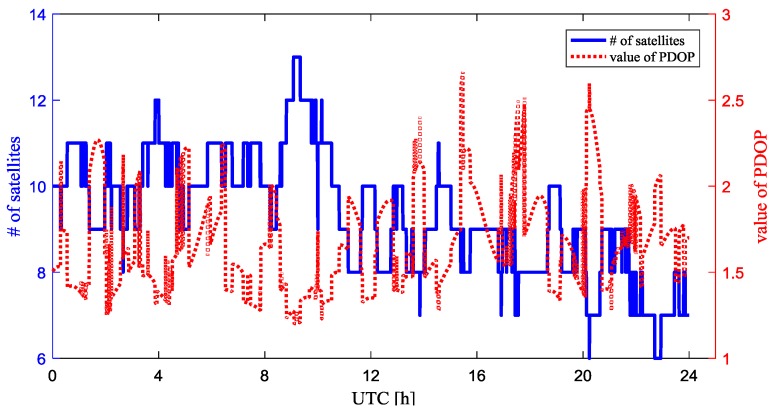
Visible GPS satellites number and PDOP value.

To simulate the moving base station scenario, we assume that the absolute position of CUT00, CUTB0, CUTC0, CUTA0 are unknown, given the baseline lengths. We set CUT00 as the rover. Figure 3 shows the number of the visible GPS satellites and the positional dilution of precision (PDOP) value for baseline between rover and the base station during the experiment, which helps to evaluate the observing conditions. The cutoff elevation angle is 5 degree. The number of visible GPS satellites varies from 7 to 13, while the PDOP values were smaller than 2 at most times.

**Figure 4:**
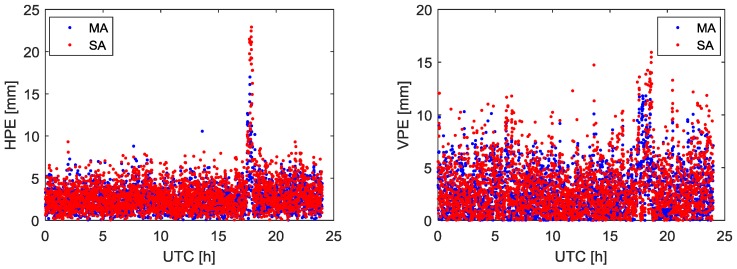
Relative position estimation errors using MA and SA. The (**Left**) panel is horizontal position estimation error. The (**Right**) panel is vertical position estimation error.

With the baseline lengths shown in Table 1, the fused code and phase measurements at the virtual point can be calculated as the measurements of base station. We can get the GPS double difference (DD) relative position estimation between the virtual point and the rover. The estimation accuracy and precision are shown in Figure 4 and Table 2.

The experiment initially demonstrates the feasibility of the multiple moving receiver data fusion method. Considering all receivers of the base station and rover used in this experiment are installed at the top of the building in an open field of vision, the number of GPS satellites received and satellite geometry is appropriate. In addition, the baseline between the reference point and rover can be considered a short baseline. Therefore, in Figure 4, the difference in position estimation accuracy obtained by using MA and the SA is not obvious. In Table 2, the position estimation precision using MA and the SA is at the same level, and the ambiguity resolution success rates are all 100%.

**Figure 5:**
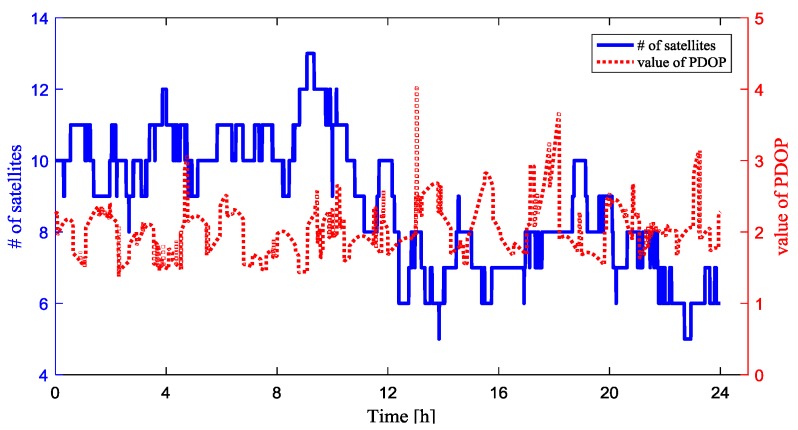
Visible GPS satellites number and PDOP value.

To test the relative positioning performance with a longer baseline distance, we use PERT as the rover instead of CUT00. PERT is a permanent GNSS reference station in Australia in the IGS network, which is equipped with a TRM59800.00 antenna and TRIMBLE NETR9 receiver. The distance between PERT and the virtual point is about 22.4 km. To mitigate the effect of DD measurement residual errors, we increased the elevation angle to 10 degrees for the long baseline test. The visible GPS satellites and the PDOP value for baseline between rover and the base station are shown in Figure 5, with a cutoff elevation angle of 10 degrees. During the experiment, 5 to 13 satellites were observed at the rover station. The PDOP values were oscillating near the value of 2 most of the time.

**Figure 6:**
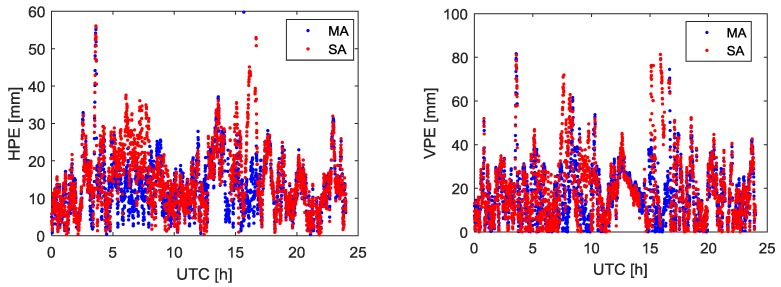
Relative position estimation errors using MA and SA. The (**Left**) panel is horizontal position estimation error. The (**Right**) panel is vertical position estimation error.

With the same fused code and phase measurements at the virtual point, the relative position estimation accuracy and precision are shown in Figure 6 and Table 3.

The relative position estimation errors in Figure 6 are increased compared with Figure 4 for both horizontal and vertical components. The position accuracy of MA solution is comparable with the SA solution. When compared with the SA, it can found that the ambiguity resolution success rate of MA is increased by 3.06%. Furthermore, the relative position estimation precision can be reduced by 15.9% for the horizontal component and 15.7% for the vertical component, compared with SA solution shown in Table 3. The relative position estimation accuracy decreases mainly due to the de-correlation of the ionospheric and tropospheric delays with growing rover to reference point distance. MA solution can obtain better quality measurements and then have better ambiguity resolution success rate and position estimation precision compared with the SA solution. This is mainly because the MA solution pulls down the errors and generates higher precision fused measurements than the individual measurement used in SA solution.

The fused measurements obtained using baseline length in ECEF frame directly are not affected by the platform baseline errors and the attitude errors. However, if the baseline length in ECEF frame is unknown, it needs to be calculated by baseline length in body frame and attitude in navigation frame. In this case, the uncertainty of fused measurements is influenced by platform baseline errors and attitude errors, as shown in Equation (15). We use the static experiment data to evaluate the effect of attitude errors and baseline errors on relative position estimation results. As all the reference receivers are static to simplify simulation, the body frame and the navigation frame are assumed to be coincident. Different error models including baseline errors and attitude errors are used in the simulation.

**Figure 7:**
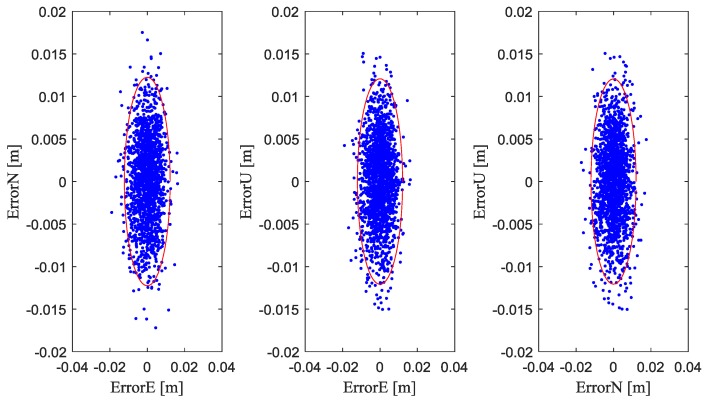
Baseline-induced relative position estimation errors. The (**Left**) panel is the errors in east and north direction. The (**Middle**) panel is the errors in east and up direction. The (**Right**) panel is the errors in north and up direction.

${\hat{b}}_{Rr,b}$ is estimated as the baseline vector in the body frame, which can be obtained by platform survey or estimation in real time. Any estimation process for baseline vector cannot have a perfect solution. Therefore, there will be a residual uncertainty of ${\hat{b}}_{Rr,b}$. We assume the uncertainty of baseline has a standard deviation of 1 cm [37] in the simulation. Figure 7 and Table 4 shows the baseline-induced extra position estimation errors with fused measurements. In Figure 7, the cloud of points represents the position estimation errors caused by baseline errors. The red ellipses represent 95% of position estimation errors. From Figure 7 and Table 4, it is seen that baseline uncertainty with a standard deviation of 1 cm causes centimeter-level accuracy changes in position estimation accuracy.

For attitude errors, it is also difficult to specify values as the expected errors in the simulation. This is because the attitude errors mainly depend on grade of attitude sensors. To show the effect of attitude errors on the final position estimation result, we assume that the standard deviation of attitude errors is approximately 0.05° to 0.3° in the simulation [38,39,40,41].

**Figure 8:**
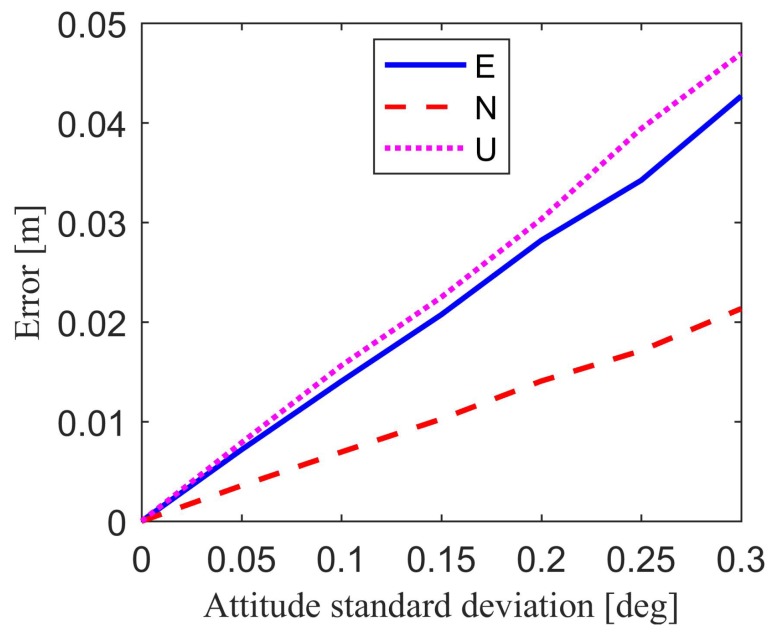
Attitude-induced position estimation errors.

**Figure 9:**
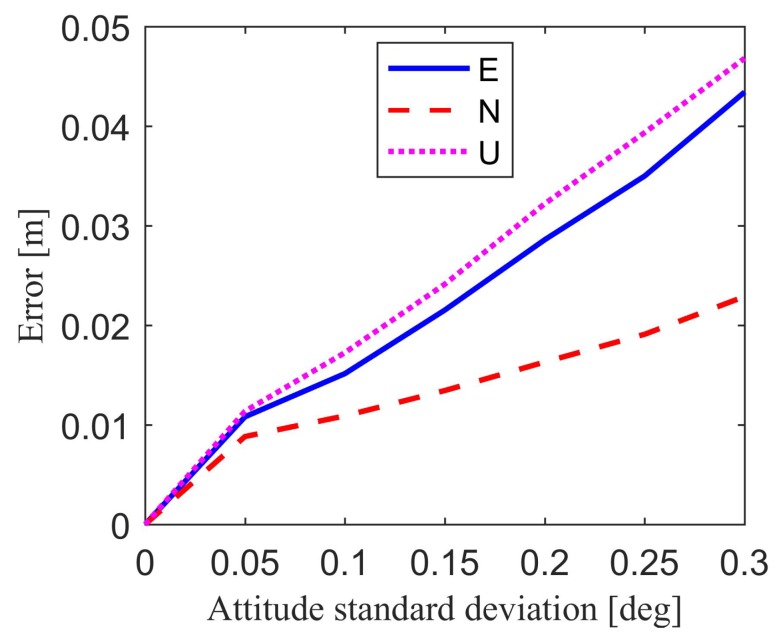
Combined error-induced position estimation errors.

Figure 8 and Table 5 show the relative position estimation errors in east, north, up component, and position estimation precision caused by attitude errors. It is clear that if the attitude standard deviation is small enough, for example smaller than 0.05°, the attitude-induced relative position estimation errors will change in millimeter-level compared with the results without attitude error. Figure 9 and Table 5 show the position estimation error due to the combined effect of baseline errors and attitude errors. The baseline standard deviation is set to 1 cm, while the attitude standard deviation ranges from 0.05° to 0.3°. If the baseline standard deviation is restricted to 1 cm, the combined baseline and attitude-induced position estimation errors have far less difference compared with attitude-induced position estimation errors. This means that the baseline length error with the standard deviation of 1 cm has little effect on the position estimation errors compared with the attitude errors. These figures and tables show the influence of the attitude errors and baseline errors on the position estimation results, thereby providing a reference for the accuracy of baseline and attitude.

### 3.2. Kinematic Experiment

**Figure 10:**
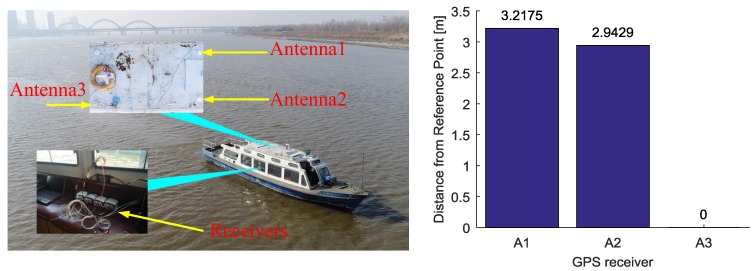
Distribution of multiple receivers and antennas. The (**Left**) panel is mounted receivers and antennas. The (**Right**) panel is baseline between the antennas and the selected virtual point.

The kinematic experiment was conducted on the Songhua River of Harbin, China. The receiver antennas are mounted on the roof of the ship, as shown by Figure 10. Three reference receivers (BDM683, Unicorecomm, Beijing, China) are placed in the cabin and connect with two CHCNAV A230GRB antennas and a Novatel GPS-703-GGG antenna, marked as A1, A2, A3, respectively. We use the post-processing software to obtain the coordinate of antennas A1~A3 as the benchmark. We set the receivers RCV1, RCV2, RCV3, which are connected with three antennas A1, A2, A3 respectively, as the reference receivers and set the location of antennas A3 as the reference point (R). The details of the data are given in Table 6. The cutoff elevation angle is set up to 5 degrees.

**Figure 11:**
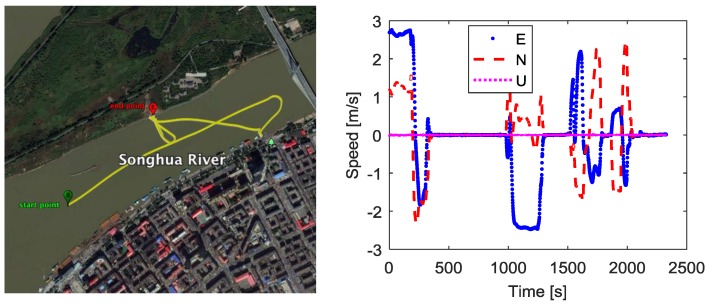
Ship’s trajectory and velocity during the kinematic experiment. The (**Left**) panel is trajectory. The (**Right**) panel is velocity.

In the kinematic experiment, the ship sailed on the river for about 39 min. The horizontal trajectory is shown in Figure 11, and the velocity of the ship are shown in the right part of Figure 11. The ship moved irregularly during the experiment, and the three-dimensional velocity ranged from 0 m/s to 3 m/s.

**Figure 12:**
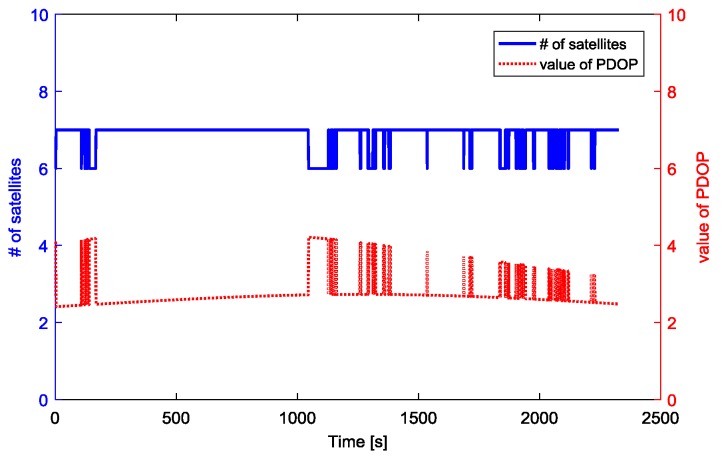
Visible GPS satellites number and PDOP value.

**Figure 13:**
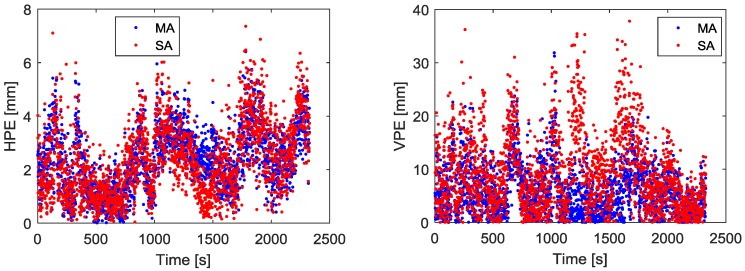
Relative position estimation errors using MA and SA. The (**Left**) panel is horizontal position estimation error. The (**Right**) panel is vertical position estimation error.

We use the fused measurements at virtual R as the measurements of the base station. The fused measurements are obtained by the measurements from three moving receivers RCV1, RCV2, RCV3 which are connected with three antennas A1, A2, A3 respectively. One receiver located at Harbin Engineering University, China, is used as the rover. The distance between the rover and start point in Figure 11 is about 4.1 km. During the kinematic experiment, the number of visible GPS satellites and PDOP values for baseline between rover and the base station are shown as Figure 12. The visible GPS satellites number varied from 6 to 7, while the PDOP values were lager than 2. With the fused code and phase measurements at virtual point, the GPS DD relative position estimation accuracy and precision are shown in Figure 13 and Table 7.

Figure 13 gives the final relative position estimation accuracy of the kinematic experiment, from which we can see that the position estimation accuracy using MA and the SA is at the same level after ambiguity resolution. However, the ambiguity resolution success rate using MA is increased by 1.33%, which is obtained with a cutoff elevation angle of 5; the position error can be reduced by 26.9% for vertical component when compared with SA solution, as shown in Table 7. Compared with SA solution, the MA solution can reduce the errors of the measurement. Multipath is the dominant error in short-baseline differential GPS systems. The observation data at low elevation angle is more severely affected by multipath noise. MA can mitigate multipath noise to some extent, so it got a better ambiguity resolution rate than SA.

## 4. Conclusions

In this paper, we use moving multiple reference receivers-based RTK for the real time precise relative positioning. To reduce the communication burden and improve the relative positioning accuracy, we have developed a code and phase measurements fusion method to combine GPS measurements from multiple receivers mounted on a moving platform for real time relative positioning. The proposed method takes advantage of known antenna geometry, integer ambiguity transformation, and error absorption, as well as similar ionospheric and tropospheric delays, to generate the fused measurements.

The presented static and kinematic experiment results were based on single epoch double difference positioning with a moving base station. In the static experiment, when the baseline is increased to 22.4 km, the ambiguity resolution success rate is increased by 3.06%, and the relative position estimation precision is reduced by 15.9% for the horizontal component and by 15.7% for the the vertical component compared with the SA solution. In the kinematic experiment, the ambiguity resolution success rate is increased by 1.33%, and the relative position estimation precision is reduced by 26.9% for the vertical component, compared with the SA solution. Along with the relative positioning performance test, we also evaluated the baseline-induced, attitude-induced, combined baseline and attitude-induced relative position estimation errors in the MA solution with different error models in the simulation. The results showed that if the attitude standard deviation is smaller than 0.05° and the baseline standard deviation is restricted to 1 cm, the 95% horizontal and vertical relative position estimation precision bounds are less than 0.0169 m and 0.0137 m respectively.

The proposed approach is suitable for applications which have multipath noise and require high real time performance. Since the measurements fusion model can convert the measurements of receivers distributed in different locations on the platform to measurements at any point on the platform and obtain the relative position between the point and the user, it can be used in applications that need to determine the relative position of a given point where it is inconvenient to install the GPS receiver.

## Figures and Tables

**Table 1:** Details of data in static experiment.

Station	Date	Rover/Base	Baseline (to R)	Interval	Duration
CUT00	19 April 2017	Base 1	8.42 m	30 s	24 h
CUTB0	19 April 2017	Base 2	9.47 m	30 s	24 h
CUTC0	19 April 2017	Base 3	5.75 m	30 s	24 h
CUTA0	19 April 2017	the virtual point (R)	0 m	30 s	24 h

**Table 2:** Relative position estimation precision and ambiguity success rate.

User	σ_H,95%_ (cm)	σ_V,95%_ (cm)	Ambiguity Success Rate
MA	0.61	0.68	100%
SA	0.75	0.81	100%

**Table 3:** Relative position estimation precision and ambiguity success rate.

User	σ_H,95%_ (cm)	σ_V,95%_ (cm)	Ambiguity Success Rate
MA	2.90	3.93	96.98%
SA	3.45	4.66	93.92%

**Table 4:** Baseline–induced relative position estimation precision and ambiguity success rate.

Standard Deviation	σ_H,95%_ (cm)	σ_V,95%_ (cm)	Ambiguity Success Rate
1 cm	1.41	0.97	100%

**Table 5:** Attitude-induced and combined error-induced relative position estimation precision.

Standard Deviation	Attitude-Induced Only		Combined Error-Induced	
	σ_H,95%_ (cm)	σ_V,95%_ (cm)	σ_H,95%_ (cm)	σ_V,95%_ (cm)
0.05°	0.94	0.94	1.69	1.37
0.1°	1.88	1.84	2.32	2.08
0.15°	2.79	2.78	3.11	2.95
0.2°	3.92	3.72	4.12	3.84
0.25°	4.71	4.72	4.89	4.80
0.3°	5.80	5.62	5.84	5.56

**Table 6:** Details of data in kinematic experiment.

Station	Date	User/Base	Baseline (to R)	Interval	Duration
RCV1	13 November 2017	Base 1	3.22 m	1 s	38.78 min
RCV2	13 November 2017	Base 2	2.94 m	1 s	38.78 min
RCV3	13 November 2017	Base 3	0 m	1 s	38.78 min

**Table 7:** Relative position estimation precision and ambiguity success rate.

User	σ_H,95%_ (cm)	σ_V,95%_ (cm)	Ambiguity Success Rate
MA	0.45	1.28	100%
SA	0.50	1.75	98.67%
